# Suppressed Expression of Allergenic Transcripts in Different Tomato Cultivars Are Linked with Increased Antioxidant Capacity

**DOI:** 10.3390/ijms26199446

**Published:** 2025-09-27

**Authors:** Katarzyna Włodarczyk, Egli C. Georgiadou, Iwona Majak, Beata Smolińska, Vasileios Fotopoulos

**Affiliations:** 1 Institute of Natural Products and Cosmetics, Faculty of Biotechnology and Food Sciences, Lodz University of Technology, 4/10 Stefanowskiego Str., 90-924 Łódź, Poland; 2 Department of Agricultural Sciences, Biotechnology and Food Science, Cyprus University of Technology, 3603 Limassol, Cyprus; egli.georgiadou@cut.ac.cy (E.C.G.); vassilis.fotopoulos@cut.ac.cy (V.F.); 3 Institute of Food Technology and Analysis, Faculty of Biotechnology and Food Sciences, Lodz University of Technology, 4/10 Stefanowskiego Str., 90-924 Łódź, Poland

**Keywords:** plant food allergens, tomatoes, gene expression analysis, ELISA assay

## Abstract

Food allergies are an increasing concern in modern society. Tomatoes as an important component of a healthy diet, are being rich in vitamins and antioxidants; however, they also contain allergens that trigger allergic reactions in approximately 2–10% of the European population. This study aimed to analyze and compare the levels of selected allergens and antioxidants in eight different tomato cultivars (four Polish and four Cypriot). Tomato cultivars were selected due to the widespread cultivation and clear differences in fruit traits and biochemical composition. This diversity provided a solid basis for examining variation in allergen expression and antioxidant content, ensuring that the quantitative real-time PCR (RT-qPCR) assay would be broadly applicable. The RT-qPCR assay successfully detected tomato allergens, while the obtained results demonstrated that Polish cultivars exhibited higher acidity, with lycopene and ß-carotene levels varying among all cultivars (*p* < 0.05). Polish cultivars contained significantly more ß-carotene than Cypriot cultivars (*p* < 0.05). Antioxidant activity, measured by ferric reducing antioxidant power (FRAP) and 2,2′-azino-bis(3- ethylbenzothiazoline-6-sulphonic acid) (ABTS) assays, revealed that certain Cypriot cultivars displayed higher antioxidant activity (*p* < 0.05), whereas Polish cultivars exhibited greater variability in antioxidant parameters. Furthermore, statistical analysis of the relationship between allergen concentration and antioxidant activity revealed distinct patterns in Polish and Cypriot cultivars. In Polish tomatoes, a strong positive correlation between antioxidant measures and allergen content was observed, while in Cypriot cultivars, the correlations between antioxidant parameters were less consistent.

## 1. Introduction

Modern society faces multiple problems associated with health concerns and the comfort of everyday life. Those difficulties and health problems are often caused by “civilization diseases” such as obesity, diabetes, cardiovascular diseases, and cancer, as well as autoimmune diseases [1,2]. One of the important public health concerns, according to the World Health Organization (WHO) [3], is food allergy. The National Institute of Allergy and Infectious Diseases (USA) describe food allergy as “an adverse reaction to a specific food antigen, which is mediated by immunological mechanisms and arises in an individual susceptible to that specific allergen [4]. Although the data concerning the prevalence of food allergy is not precise, it is estimated that it may affect approximately 5% of adults and 8–10% of children [5,6]. The majority of allergic reactions are triggered by eight major food groups—milk, eggs, fish, crustaceans, peanuts, tree nuts, wheat, and soybeans—but other foods, including fruits and vegetables, are also clinically relevant.

Tomato is one of the most widely consumed vegetables worldwide and represents a significant source of vitamins, minerals, and antioxidants [7,8,9]. However, it can also trigger allergic reactions in 1.7–9.3% of the European population, with a higher prevalence reported in Mediterranean countries [10,11,12]. Tomato allergy is primarily immunoglobulin E (IgE)-mediated, often associated with cross-reactivity to pollen allergens. Clinical symptoms usually manifest as oral allergy syndrome (OAS), characterized by itching or swelling in the oral cavity and pharynx, but gastrointestinal and systemic reactions may also occur in sensitized patients [12,13,14]. Symptoms are most often elicited by fresh tomato fruits, whereas processed tomato products are generally better tolerated. According to the WHO/IUIS Allergen Nomenclature database, seven allergens have been identified in tomato: profilin (*Sol l 1*), β-fructofuranosidase (*Sol l 2*), lipid transfer proteins (*Sol l 3*, *Sol l 6*, *Sol l 7*), pathogenesis-related protein (*Sol l 4*), and cyclophilin (*Sol l 5*). Additional IgE-binding proteins have also been reported.

Research on *Solanum lycopersicum* allergens has revealed a complex interplay between genetic, molecular, and environmental determinants shaping allergenic potential. Early investigations demonstrated that the introduction of genomic segments from wild relatives such as *S. pennellii* into cultivated tomato backgrounds significantly modifies IgE-binding patterns, particularly in relation to polygalacturonase and pectin methylesterase, two of the most relevant tomato allergens [15]. These results were expanded by studies showing that allergenicity is not only influenced by genetic background but also by cultivation conditions. In this context, Welter et al. (2013) [16] identified considerable interindividual variation in allergic responses, while also reporting novel putative allergens including endo-β-mannanase, pectinacetylesterase, and aspartyl protease family proteins, thus broadening the allergen spectrum associated with tomato.

At the molecular level, further characterization of allergen families has been achieved through genomic and transcriptomic approaches. D’Agostino et al. (2019) [17] carried out a systematic survey of the non-specific lipid transfer protein (nsLTP) gene family in tomato, identifying 64 members and confirming the biochemical relevance of *Sola l 3* as a key allergen highly expressed in fruit tissues. Clinical profiling across multiple commercial varieties corroborated the importance of LTPs, as well as polygalacturonase isoforms such as PG2A, with significant variability observed among cultivars, including heightened IgE reactivity in the Kumato variety [18]. Beyond these major allergens, profilin (*Sola l 1*) has also been recognized as an important sensitizer. Kiyota et al. (2017) [19] demonstrated its presence not only in fresh tomatoes but also in processed tomato products, noting that its IgE-binding activity remains stable after heat or enzymatic treatment. More recently, in silico analyses of thaumatin-like proteins, particularly NP24, revealed significant epitope similarities and potential cross-reactivity with allergens from related and unrelated plant species such as capsicum, olive, kiwi, and banana, suggesting broader immunological implications for tomato allergy [20].

Given the clinical relevance of tomato allergy and the legal requirement for allergen labelling in food products, reliable detection methods are essential. The enzyme-linked immunosorbent assay (ELISA) remains the most widely used technique due to its high sensitivity, specificity, and relative simplicity [21]. However, ELISA has limitations, including cross-reactivity caused by sequence homology between allergenic proteins, susceptibility to matrix effects, and decreased reliability when proteins are degraded during food processing. In addition, antibody instability and the cost of production further constrain its application.

The alternative for immunoassays may be DNA-based methods, such as real-time polymerase chain reaction (qPCR). Firstly, DNA molecules maintain their integrity better than proteins [21] which excludes the problem with processed food and furthermore DNA-based tests are less affected by matrix effects than ELISA. Moreover, to conduct PCR reactions the use of specific primers, probes and reagents is required, which allows to carry out the specific and very sensitive reactions. Due to this specificity and the fact that this method is based on DNA/cDNA sequence, there is no problem with cross-reactivity. The fact that DNA sequences can enhance the specificity of the assay, it is easier to identify DNA sections that lack homology with other species. Nonetheless, experimental specificity testing is necessary to confirm that the primers intended for a certain species do not bind to a non-target species. So far, several studies have been conducted which employed real-time PCR tools for detecting allergens [22,23]. For instance, Watanabe et al. (2012) [24] have developed two PCR methods for peach (*Prunus persica*) and apple (*Malus domestica*) allergen detection. The method was based on PCR amplification followed by electrophoresis on agarose gel. The sensitivity of this method reached approximately 10 ppm of the ingredient. The use of PCR methods in allergen detection has been well developed in the field of tree nut allergens [25]. In the study of Köppel et al. (2009) [26], two tetraplex real-time PCR methods were developed. The procedures proposed by Köppel et al. [26] allow simultaneous detection of peanut, hazelnut, celery, and soy in one multiplex reaction. Additionally, detection of egg, milk, almond, and sesame in a second established reaction was possible. In recent years, the use of real-time PCR technologies and digital PCR has significantly escalated. Suh et al. (2019) [27] employed a multiplex PCR technique to identify possible fruit allergens responsible for food allergy labelling in Korea. Targeted primer pairs were developed to amplify the allergen-coding genes Cyclophilin (tomato), Mdtl 1 (apple), Pru p 2.01A (peach), and Pectin methylesterase inhibitor (kiwi). This test is anticipated to be a precise and effective technique for identifying fruit allergens in food products.

The present work reports the development of real-time RT-qPCR assay encoding genes encoding chosen tomato allergens (*Sola l 1*, *Sola l 2*, *Sola l 3*, *Sola l 4*, *Sola l 5*, *Sola l 6*) which was used to compare the amount of several tomato allergens. These six allergens were selected due to their established or presumed involvement in triggering IgE-mediated hypersensitivity responses in susceptible individuals [28,29]. Monitoring the expression of these allergen-related genes was considered crucial for consumer safety, as their presence and abundance could influence the allergic potential of tomato-based products. Furthermore, accurate detection and quantification supported compliance with food labelling regulations, enabling consumers to make informed choices and reducing the risk of unintendedly allergen exposure. The obtained data will allow us to analyze the relationship between allergen levels and the activity or concentration of selected antioxidants in the examined tomato fruits. The reaction efficiency and detection sensitivity of the RT-qPCR method were evaluated, and the developed assay was shown to have potential as an improved reference analytical procedure for tomato allergen detection. The application of RT-qPCR in the present study addressed a clear methodological gap in allergen detection in tomato. Until now, allergen quantification in this species has relied primarily on ELISA-based immunoassays (e.g., for *Sola l 1* and *Sola l 4*), multiplex PCR approaches targeting allergen-related genes, or, more recently, PCR-free electrochemical biosensors (e.g., for *Sola l 7*). However, none of these techniques provide the same combination of sensitivity, specificity, and robustness as nucleic acid–based real-time quantification. By implementing RT-qPCR for allergen-encoding genes in tomato cultivars, this study introduces a more reliable and reproducible strategy that overcomes the limitations of protein instability, cross-reactivity, and methodological inconsistency inherent in existing approaches. The use of both methods is complementary: ELISA allows detection of the actual protein products and provides direct information about allergen presence, while qRT-PCR enables a broader assessment of gene expression even in cases where antibody-based detection is not feasible. In addition, this research employed and compared the results of the developed method with those obtained from ELISAs and measurements of antioxidant activity in the tested tomato fruits. This comparison enabled the assessment of possible association between allergen content and antioxidant activity.

This work was innovative as it combines molecular allergen detection, nutritional profiling, and cross-regional cultivar comparison to identify new correlations between tomato antioxidants and allergens, with direct impactions for consumer safety, plant breeding, and food labelling.

## 2. Results

### 2.1. Measurements of Titratable Acidity (TA)

Results obtained in titratable acidity (TA), measured in tomatoes, exhibited the specific trend (Table 1). TA in Polish fruits ranged between 0.039 and 0.087 g/100 g DW., which was significantly higher compared with the acidity levels detected in Cypriot fruits. Cultivar F179 was the only Cypriot cultivar with TA value comparable to that of Polish fruits.

### 2.2. Examination of Lycopene and β-Carotene Content

**Figure 1:**
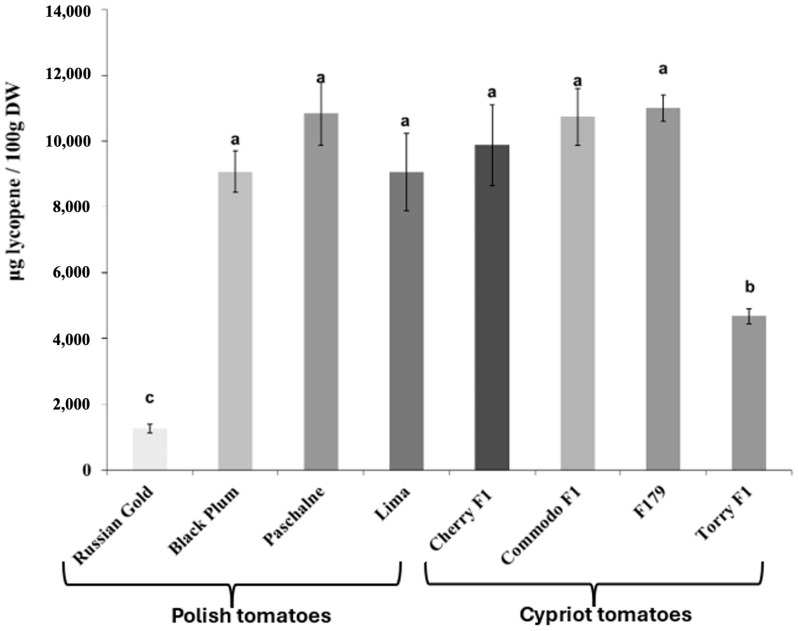
Concentration of lycopene in tomato cultivars of Polish tomatoes (Russian Gold, Black Plum, Paschalne, Lima) and Cypriot tomatoes (Cherry F1, Commodo F1, F179, Torry F1). Different letters indicate statistically significant differences (*p* < 0.05) when analyzed using Duncan’s multiple range test. Data represents the means of three biological replications ± SE.

**Figure 2:**
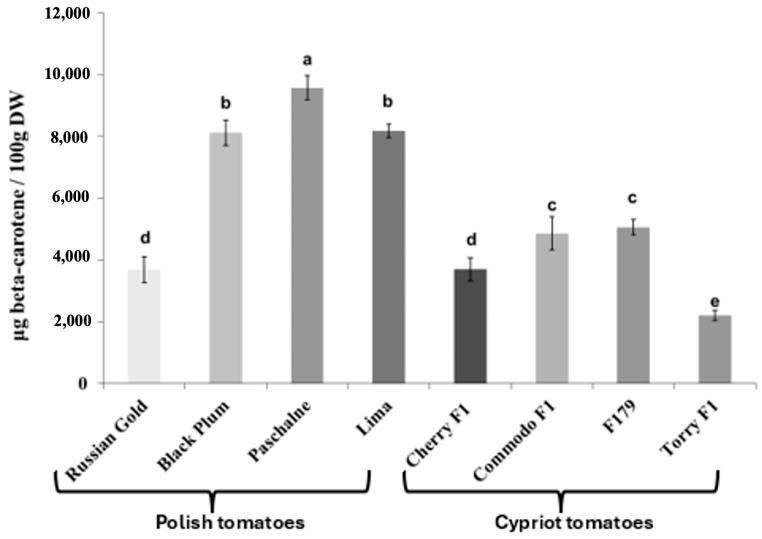
Concentration of β-carotene in tomato cultivars of Polish tomatoes (Russian Gold, Black Plum, Paschalne, Lima) and Cypriot tomatoes (Cherry F1, Commodo F1, F179, Torry F1). Different letters indicate statistically significant differences (*p* < 0.05) when analyzed using Duncan’s multiple range test. Data represents the means of three biological replications ± SE.

The determination of lycopene and β-carotene content in all examined cultivars yielded variable results. Figure 1 illustrates that most cultivars exhibited similar levels of lycopene content. Those cultivars were BP, PAS, Lima, CH, COM and F179 and they reached a lycopene content at 9.1–11.0 mg/100 g DW (on average 10.1 mg/100 g DW). Nevertheless, two cultivars (RG, TOR) had significantly lower lycopene content compared with others. The lycopene content was lower by 87.5% in RG and by 54.0% in TOR in relation to average value for the other cultivars, The examination of β-carotene content exhibited a large degree of variation between cultivars (Figure 2). A notable difference was observed between Polish and Cypriot cultivars, with the latter exhibiting lower β-carotene content compared to the Polish ones. The lowest β-carotene content was recorded in TOR cultivar and was equal 2.2 mg/100 g DW). Cultivars COM and F179 reached similar values, varying between 4.8 and 5.1 mg/100 g DW. Cherry F1 presented a β-carotene content lower than COM and F179 by approximately 25.6% and increased by 67.7% in comparison with TOR. Among Polish cultivars, only RG displayed similar β-carotene levels to Cypriot ones. Contrarily, the three other Polish cultivars (BP, PAS and Lima) exhibited significantly higher β-carotene content than RG. The highest β-carotene amount was recorded in cultivar PAS, with a value equal to 9.6 mg/100 g DW.

### 2.3. Antioxidant Capacity of Tomatoes

**Figure 3:**
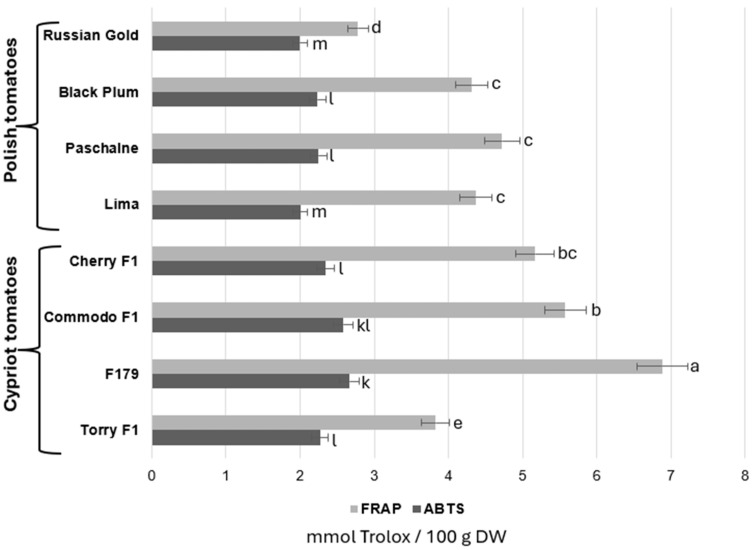
Comparison of antioxidant activity of Polish tomatoes (Russian Gold, Black Plum, Paschalne, Lima) and Cypriot tomatoes (Cherry F1, Commodo F1, F179, Torry F1) evaluated with FRAP and ABTS assays. Different letters indicate statistically significant differences (*p* < 0.05) between comparable groups: for FRAP (a–e)); for ABTS (k–m) when analyzed separately using Duncan’s multiple range test. Data represents the means of three biological replications ± SE.

The examination of general antioxidant capacity in all eight cultivars was realized with the use of two assays: Ferric Reducing/Antioxidant Power (FRAP) and the 2,2′-azino-bis (3-ethylbenzothiazoline-6-sulphonic acid) (ABTS) method. The results of these investigations were shown in Figure 3. The results obtained with the FRAP analysis showed that four tomato cultivars presented similar levels of antioxidant activity, ranging between 4.3 and 5.2 mmol/100 g DW, regardless of the cultivar origin. The lowest activity was recorded in both RG tomatoes belonging to a Polish cultivar and TOR which is a Cypriot cultivar and was as follows 2.78 and 3.83 mmol/100 g DW, respectively. In contrast, two other cultivars, COM and F179, reached much higher antioxidant activity according to FRAP analysis in relation to all examined fruits. In comparison with RG tomatoes, which achieved the lowest antioxidant activity potential overall, COM and F179 increased the antioxidant activity by 100.7% and 146.8%, respectively. The analysis of antioxidant activity measured with the ABTS assay provided similar results. The Lima cultivar demonstrated a trend of slightly reduced antioxidant activity compared to most other cultivars, though this effect was not statistically significant. Moreover, the cultivars that presented the lowest antioxidant activity according to the ABTS protocol were RG and Lima. Contrary to the results obtained by FRAP analysis, TOR cultivar demonstrated similar activity as BP, PAS and CH, ranging between 2.23 and 2.34 mmol/100 g DW. Furthermore, the highest antioxidant activity according to the ABTS assay was recorded in F179 cultivar, which, in comparison with RG fruit, increased by 34%.

### 2.4. Allergen Content by Immunoassay Analysis

**Figure 4:**
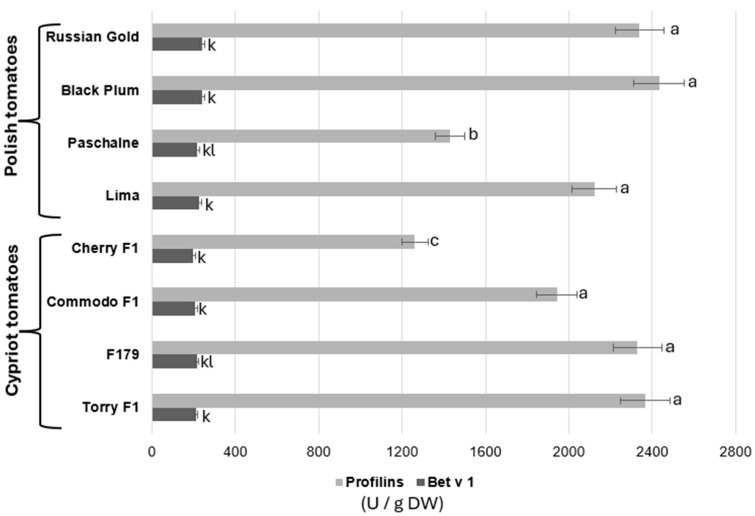
Comparison of the quantity of allergenic proteins: profilins and Bet v1 of Polish tomaatoes (Russian Gold, Black Plum, Paschalne, Lima) and Cypriot tomatoes (Cherry F1, Commodo F1, F179, Torry F1) assayed by ELISA analysis. Different letters indicate statistically significant differences (*p* < 0.05) between comparable groups: for Profilins (a–c)); for Bet v 1 (k–l) when analyzed separately using Duncan’s multiple range test. Data represents the means of three biological replications ± SE.

For comparison of methods for allergen detection, the enzyme-linked immunosorbent analysis was implemented. Results are presented in Figure 4. The analysis of profilin content in all eight tomato cultivars showed that most of examined cultivars contained similar concentration of this allergen. The profilin concentrations detected in cultivars RG, BP, Lima, COM, F179 and TOR ranged between 1942 and 2432 U/g DW, although no statistically significant differences were observed. Nonetheless, two of the cultivars, PAS and CH, presented considerably lower profilin amounts, while the highest profilin contents were recorded in PAS (1440 U/g DW) and CH (1261 U/g DW). Bet v 1 allergen examination indicated that, the concentration of this allergen varied considerably among all cultivars. The highest concentration of Bet v 1 was recorded in two Polish cultivars, RG and BP (241 U/g DW). Cultivars PAS and Lima displayed declines in Bet v 1 content by 9.4% and 5.7%, respectively, when compared to RB and BP. Cypriot cultivars showed a major decrease in Bet v 1 concentration, ranging between 199 and 209 U/g DW in relation to Polish ones.

### 2.5. Allergenic Gene Expression Profiles of Different Tomato Cultivars

Allergen-specific primers were designed after the extraction, purification, and analysis of RNA from tomato samples (homogenized flesh and peel). Supplementary Table S1 illustrates the sequence of the developed primers and the selected annealing temperatures. Designed primers are specific to allergenic protein-coding genes. The F179 tomato cultivar was employed for calibration, as this variety is an old and traditional Cypriot cultivar that was considered a reference in this research. All tomato cultivars displayed differential regulation of the examined genes. The analysis of relative gene expression levels in allergen formation was demonstrated in Supplementary Table S2. Different patterns of expression were observed in all examined tomatoes; however, only *SlSola l 1* allergen, which encodes profilin formation, was detected as being up-regulated in all cultivars. Furthermore, higher expression levels of *SlSola l 1* were observed in Polish cultivars, especially BP and PAS. When compared with the control (F179), gene expression levels observed in all Polish cultivars and COM from Cypriot varieties were significantly higher. In the case of *Sola l 2* allergen, results indicate a different pattern of expression. The expression of *SlSola l 2* was highly induced in cultivars BP, PAS and Lima compared with the control. For Cypriot tomatoes, *SlSola l 2* appeared to be significantly down-regulated. The analysis of gene expression levels encoding Sola l 3 indicates that TOR is the only cultivar in which this gene is not expressed, but this result is not significantly different compared with the control. In all other cultivars, *SlSola l 3* is significantly induced in cultivars RG, Lima and COM. In the case of the Bet v. 1 allergen *(Sola l. 4*), TOR is the only cultivar in which the relative expression level was significantly decreased in comparison to the control. For other cultivars, *SlSola l 4* was up-regulated, and the highest induced level was performed by PAS cultivars. Genes encoding *Sola l 4* in this cultivar, as well as RG, BP and COM are considerably up-regulated when compared to the control. All Polish cultivars significantly increased the expression level of *Sola l 5*.

Although two Cypriot varieties, CH and COM were up-regulated as well, those results are not considerably higher than the control. For TOR the *SlSola l 5* expression was down-regulated; however, in comparison to the control, this result is not considered significant. *Sola l 6* was the last allergen to have its expression level evaluated. The expression level of gene encoding in this allergen was not significantly changed in RG cultivar. In all other cultivars, the obtained results vary considerably from the control. The gene expression exhibited by COM and TOR cultivars was strongly down-regulated, while other cultivars displayed the opposite tendency. Cultivars BP, PAS, Lima and CH performed a significantly induced level of *SlSola l 6* gene expression when compared to the control. The highest value was presented by cultivar PAS (Supplementary Table S2).

**Figure 5:**
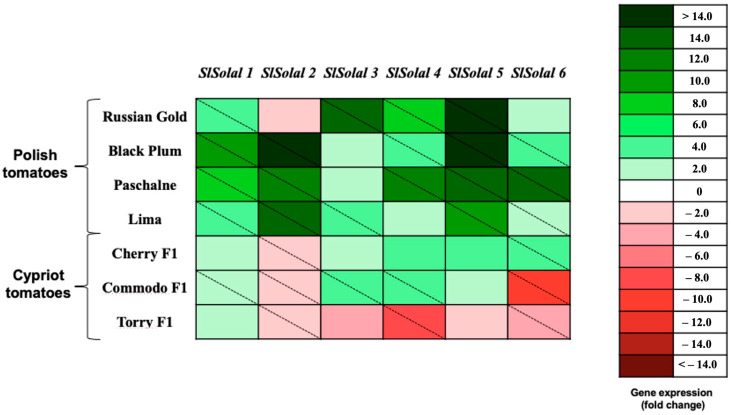
Heat map of the relative expression levels of allergenic protein transripts (*SlSolal 1*, *SlSolal 2*, *SlSolal 3*, *SlSolal 4*, *SlSolal 5* and *SlSolal 6*) of four tomato cultivars from Poland (Russian Gold, Black Plum, Paschalne, Lima) and four tomato cultivars from Cyprus (Cherry F1, Commodo F1, F179, Torry F1) (n = 3). Relative mRNA abundance was evaluated by real-time RT-qPCR using three biological repeats. Up-regulation is indicated in green; down-regulation is indicated in red. A diagonal line in a box indicates a statistically significant difference (*p* ≤ 0.05). A scale of colour intensity is presented as a legend. The tomato cultivar ‘F179’ from Cyprus was used for calibrating gene expression values. Actual relative expression levels are shown in Supplementary Table S2.

To acquire further insight into the obtained data, the results were evaluated, compared, and exhibited in the form of a heat map (Figure 5).The construction of the heatmap was motivated by the need to analyze the transcriptional patterns at the variety level, thereby providing a comparative overview of gene expression across cultivars instead of a gene-specific view. Firstly, the analysis of the presented heat map indicates that in the case of the Polish cultivar used in this research, all six allergens were up-regulated. The one exception is RG cultivar; thus, the expression gene encoding *Sola l 2* allergen was down-regulated in tissues of this cultivar. However, this result cannot be considered as significant as indicated by the statistical analysis. A similar situation may be observed for CH cultivar, which belongs to the Cypriot varieties. Nevertheless, the difference between CH and RG is that in the case of CH cultivar, the down-regulation of genes encoding *Sola l 2* is significant. Moreover, the expression of all up-regulated genes (except *SlSola l 6*) in RG cultivar is considerably higher than in CH tomatoes. Furthermore, in all Cypriot cultivars the expression of genes encoding *Sola l 2* allergen is down-regulated, while for Polish tomato cultivars it was only observed in RG. On the contrary, in Cypriot tomatoes, the expression of *SlSola l 2* is significantly suppressed. The specific tendency may be observed in tomatoes of TOR cultivar. In the case of this tomato variety, only the genes that encode the expression of *Sola l 1* are up-regulated, although this result is not significantly higher compared to the control. Other genes representing five other allergens are down-regulated.

### 2.6. Correlation Between Antioxidant Components and Allergens

**Figure 6:**
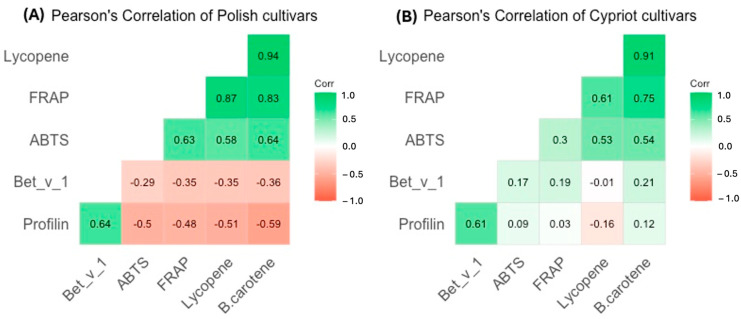
The Pearson’s correlation between the results of antioxidant and allergenic potential of (**A**) Polish tomatoes and (**B**) Cypriot tomatoes.

The correlation analysis on the selected results was conducted. The Pearson’s linear correlation coefficient was employed as the primary indicator of the strength of the linear relationship between observable characteristics. Figure 6 illustrates Pearson’s correlation analysis between the allergen concentration in tomatoes (determined using ELISAs) and the detected antioxidant activity and content in the same fruits. The analyzed fruits were divided into two groups: Polish (Figure 6A) and Cypriot cultivars (Figure 6B).

**Figure 7:**
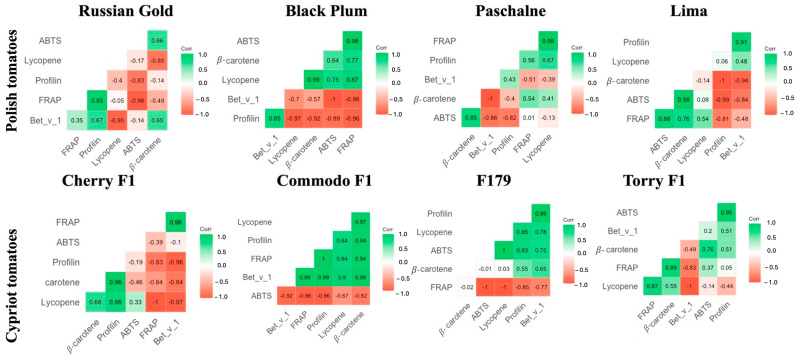
Graphic representation of Pearson’s correlation between antioxidants properties and concentration of chosen allergens of Polish tomato (Russian Gold, Black Plum, Paschalne, Lima) and Cypriot tomatoes (Cherry F1, Commodo F1, F179, Torry F1).

Based on the results it was found that FRAP, ABTS, lycopene, and β-carotene content, measured in Polish cultivars, were strongly linked with correlation coefficients, ranging from 0.58 to 0.94. The measures characterizing antioxidant activity and profilin content revealed a modest association, with the correlation varying between 0.48 and 0.59. Further examination exhibited a modest correlation between the amount of the allergen Bet v 1 and antioxidant potential indicators (Figure 7).

For Cypriot cultivars, the association between antioxidant activity parameters was low for ABTS and strong for FRAP. The correlation between allergens (both profilins and Bet v 1) and indicators of antioxidant potential was significantly lower in Cypriot cultivars than in Polish ones. A weak association between the aforementioned results was observed.

## 3. Discussion

### 3.1. Comparison of Fruit Quality

Fruit quality is determined by a wide range of attributes, including size, texture, sugar content, and acidity [30]. Among these, TA plays the crucial role in defining sensory properties, particularly flavour balance, and serves as a valuable indicator of ripeness [30]. The analysis of TA in all examined cultivars exhibited significant differences between Polish and Cypriot cultivars, which can be largely attributed to several factors. Acidity levels are strongly dependant on cultivar-specific metabolic characteristics, as different genotypes exhibit distinct capacities for organic acid accumulation. In addition, the fruit development stage influences TA, with acidity decreasing as ripening progresses, while sugar content rises. Moreover, the acidity of tomatoes can be influenced by environmental factors such as temperature, humidity, soil composition, and water availability. For instance, water deficit-induced stress is associated with an increase in acidity, reflecting adaptative physiological responses. The variations observed in TA value measured in Polish and Cypriot cultivars underline the importance of both genetic background and environmental factors influence in determination tomato acidity.

### 3.2. Antioxidant Activity

The evaluation of antioxidant capacity with FRAP and ABTS assays revealed consistent trends across the examined cultivars, indicating that the antioxidant potential of tomatoes is strongly influenced by both genetic background and growing environment. While cultivation in organic systems ensured comparable production practices, differences between Polish and Cypriot cultivars likely reflect environmental conditions such as temperature regime and rainfalls. As shown in Supplementary Table S3, Cypriot cultivars were grown at higher temperatures and lower atmospheric precipitation when compared to Polish ones, which was generally reflected in their increased antioxidant potential, regardless the method of determination. Because detailed climate datasets at the level of the specific cultivation sites were not available, national averages were used as a proxy, and we now explicitly acknowledge in the text that such data may mask regional variability. The study of Juroszek et al. (2009) [31] showed that the production system itself (organic versus conventional) does not markedly influence the level of bioactive compound content or antioxidant activity in tomatoes. However, Tilahun et al. (2018) [32] highlighted the importance of cultivar selection as a decisive factor affecting fruit quality.

Further insight in antioxidant activity of Polish and Cypriot tomatoes was obtained from the analysis of lycopene and β-carotene concentration in tomatoes fruits. Lycopene amounts were generally stable across most cultivars, with two exceptions: RG (Polish) and Torry F1 (Cypriot), which showed significantly lower lycopene levels compared to other cultivars. The role of temperature and light in regulating lycopene biosynthesis is well established, with suboptimal (below 12 °C) or extreme (above 32 °C) temperatures shown to suppress accumulation, whereas moderate light exposure promotes lycopene synthesis [33]. The comparative analysis of obtained results demonstrated that β-carotene concentrations were higher in Polish cultivars, which is consistent with the known differential responses of carotenoid biosynthetic pathways to temperature fluctuations. Moreover, the β-carotene biosynthesis pathway is strictly dependent on lycopene [28]. Specifically, elevated temperatures have been reported to inhibit lycopene accumulation while enhancing β-carotene synthesis [34].

The relationship between antioxidant composition and allergen-related gene expression provides an additional perspective on the interplay between secondary metabolism and fruit allergenicity. Reduced carotenoid and antioxidant levels were associated with down-regulation of specific allergen-encoding genes in some cultivars, like Torry F1. For this cultivar, the low activity of antioxidants and the low concentration of lycopene and β-carotene were correlated with down-regulation of all genes encoding allergens examined in those fruits. In other cultivars, the distinct expression patterns were observed, including selective up-regulation. In RG cultivar, lowering concentration and activity of selected antioxidants did not affect the expression of genes encoding all allergens. For RG tomatoes, only *Sola l 2* genes were down-regulated and on the contrary, *Sola l 3* genes were significantly up-regulated. Subsequently, the low concentration of β-carotene was furthermore correlated with down-regulation of *Sola l 2* in CH and COM tomatoes. Moreover, the increased level of the aforementioned antioxidant was also detected in Polish cultivars. These observations suggest a potential link between antioxidant profiles and allergen gene regulation. Although such associations have not been previously documented, the current findings provide a basis for further investigation, particularly into the regulation of genes involved in carotenoid biosynthesis and their interaction with allergen expression.

### 3.3. Analysis of Allergen Content

In this research, the development of an RT-qPCR assay, which is targeting the genes encoding for six allergenic proteins from tomato fruits, was provided. The obtained data of selected gene expression was followed by the immunoassay, which was provided in terms of examination of allergen detection by both conducted methods. In addition, the analysis of total antioxidant activity and the evaluation of lycopene and β-carotene were conducted to better characterize the examined tomatoes. To conduct RT-qPCR research on specific tomato allergens, eight distinct tomato cultivars were chosen for examination, including four Polish cultivars (Russian Gold (RG), Black Plum (BP), Paschalne, and Lima) and four Cypriot cultivars (Cherry F1 (CH), Commodo F1 (COM), F179, and Torry F1 (TOR)). In this study, the tomato cultivars served as samples required to standardize the developed allergen detection methodologies.

The examination of profilin (*Sola l 1*) and Bet v 1 (analogue of *Sola l 4*) content in tomato fruits was realized with the use of ELISA (enzyme-linked immunosorbent assay). The results obtained in immunoassay were not comparable with the analysis of gene expression. For instance, the *Sola l 1* expression was at a noticeably lower level in Cypriot tomatoes than in Polish tomatoes, but in the immunoassay, this trend was not observed. The differences in obtained data could be associated with the stability of the examined compound structure. The ELISA is a method that detects small amounts of proteins from specific foods. An inconvenience associated with immunoassays is that food processing can impact protein properties, potentially leading to alterations in the detection outcomes when utilizing protein-based techniques [35]. Furthermore, profilins and LTP (lipid-transfer proteins), which are abundant allergens found in tomatoes, are among the compounds that exhibit high cross-reactivity. The previously mentioned compounds are panallergens, which means they are widely distributed in nature and can cause cross-reactions in allergic patients. In the research of Asero et al. (2002) [35] the cross-reactivity of LTP proteins was proved. Patients (LTP-sensitive) experienced an adverse reaction even after ingestion of botanically unrelated plant-derived foods. The findings obtained in this study led to the conclusion that DNA/RNA-based techniques, including RT-PCR, should be suggested as a precise, sensitive, and dependable substitute for enzyme-linked immunosorbent assay (ELISA), as DNA and RNA molecules exhibit superior stability compared to proteins. Additional research is required to implement these findings in an industrial context. It is recommended to create the recombinant plasmids, which contain the target region of the aforementioned genes evaluated as a point of reference. A collaborative study for method validation would be required to determine the efficacy and suitability of the analytical method for its designated objectives.

### 3.4. Correlation Between Antioxindants Activity and Allergens Conten

Recently, several studies have investigated the relationship between the presence and abundance of allergens in plants, their antioxidant potential, and environmental factors. The statistical analysis provided in our research to determine the correlation between the two abovementioned factors (allergen content and antioxidants) showed a modest association. It is crucial to acknowledge that our study focused solely on a few parameters that characterize the antioxidant capacity of fruits, like the overall antioxidant activity and the levels of lycopene and β-carotene. The extended analysis of selected antioxidants could bring other observations. There are some studies that indicate a correlation between the content of allergens and the activity of specific antioxidants or factors involved in their biosynthesis. For example, Kitagawa et al. (2006) [36] discovered that compared to the wild-type fruit, the rin hybrid fruit (a mutant tomato with a ripening inhibitor gene) had much lower levels of genes that code for proteins that could be allergenic. The findings suggest a link between the ripening process and the development of allergic proteins. The rin mutant contains a mutation in the LeMADS-RIN gene, which encodes a MADS-box transcription factor. The MADS-box transcription factor is essential for regulating the expression of several genes associated with the ripening process, including those involved in ethylene synthesis, cell wall modification, and pigment accumulation. The LeMADS-RIN gene plays a critical role in the transformation of tomato fruits from an immature to a mature stage, exerting an influence on characteristics such as texture, flavour, and colour [36]. The authors of this study indicated that the rin mutant gene had an impact on the accumulation of allergenic proteins in tomato fruits. The other researchers found reduced quantities of the allergenic proteins β-fructofuranosidase and polygalacturonase 2A (PG-2A) in the ripening stage of the rin hybrid fruit compared to the wild-type fruit [36]. Ripening of tomatoes is closely associated with the accumulation of various antioxidants in the fruit. Throughout the ripening process, tomatoes have notable biochemical transformations that improve their nutritional and sensory characteristics. These changes result in the formation of beneficial substances, including antioxidants such as carotenoids (like lycopene and β-carotene), flavonoids, and vitamin C. Hjernø et al. (2006) [37] found a link between the low levels of allergen Bet v 1 in strawberries (*Fragaria ananassa*) and the slowing down of several enzymes that make flavonoids more abundant. The authors conducted proteomic screening of red and white strawberry mutants (*Fragaria ananassa*) by combining MALDI-MS/MS de novo sequencing of double-derivatized peptides with indel-tolerant searching against local protein databases containing extracted sequence tags (EST) and full-length nucleotide sequences. White strawberries were examined due to their reputation as being well-tolerated by individuals with allergies. A comparison between red strawberries and white mutants revealed a significant down-regulation of the Bet v-1 homologous allergen in white strawberries. Moreover, white mutants showed reduced expression of chalcone synthase, dihydroflavonol reductase, flavanone 3-hydroxylase, and methyltransferase. These enzymes are part of the well-studied flavonoid biosynthesis pathway, which also generates other chemicals such as pelargonidin, the red pigment found in strawberries [37]. The authors hypothesized that the decrease in the strawberry allergen and the production of flavonoids independently support the idea that Bet v-1 acts as a carrier of hydrophobic chemicals [38]. This hypothesis was supported by in vitro binding studies that replaced ANS (8-anilino-1-naphtalenesulphonic acid) with hydrophobic ligands like flavone and naringenin to purified Bet v 1-protein. The 45% sequence identity between Bet v 1 and Hyp-1, which is the enzyme responsible for producing hypericin, a red-coloured naphthodianthrone found in Hypericum perforatum, provides additional evidence for the involvement of the flavonoid synthesis pathway [39].

Based on the observed results, a critical distinction must be drawn between the statistical and biological significance of the correlations between antioxidant components and allergen content. The statistical analysis revealed a modest to strong linear association between antioxidant activity (FRAP, ABTS) and carotenoid content (lycopene, β-carotene) within the Polish cultivars, with Pearson coefficients as high as 0.94, indicating a statistically robust relationship within that specific sample set. Furthermore, a modest statistical correlation (r = 0.48–0.59) was observed between these antioxidant measures and profilin content in the same group. However, the biological significance of these correlations remains uncertain. The markedly weaker or non-existent correlations within the Cypriot cultivars suggest that these statistical relationships are not consistent across different genetic backgrounds and are likely influenced by other confounding variables, such as cultivar-specific genetic regulation or environmental growing conditions. This discrepancy implies that any potential connection is not direct but is instead mediated through complex, shared biosynthetic pathways, such as those governing fruit ripening—a process coordinated by transcription factors like LeMADS-RIN that concurrently influence pigment accumulation and, as noted in other studies, the expression of allergenic proteins. Therefore, while the statistical correlations in the Polish group are mathematically valid, their biological significance is questionable without evidence of a mechanistic link. They may be co-occurring phenomena rather than a direct causal relationship, underscoring the necessity for extended research into the molecular mechanisms, such as the role of allergens as carriers for flavonoid compounds, to determine if a functionally significant biological interaction exists.

## 4. Materials and Methods

### 4.1. Plant Material

**Figure 8:**
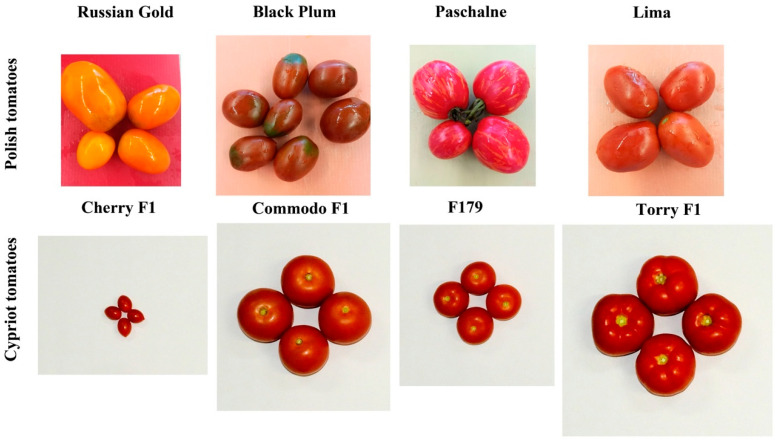
Phenotypic representation of Polish tomatoes (Russian Gold, Black Plum, Paschalne, Lima) and Cypriot tomatoes (Cherry F1, Commodo F1, F179, Torry F1) at fully ripe stage.

Genetic material used in this study included eight tomato (*Solanum lycopersicum* L.) cultivars from local farmers. Four cultivars were obtained from Polish ecological farm (Russian Gold, Paschalne, Black Plum and Lima) called “Domowa Spiżarnia” located in Lubomierz (Lwówek Śląski County, Lower Silesian Voivodeship) (N 50°59.8601′ E 15°33.7595′), while four other commercial varieties were imported from local Cypriot farmers (Cherry F1, Commodo F1, F179, Torry F1) located in Limassol district, Cyprus (34°55′37.0” N 32°58′12.0” E). The selected cultivars are characteristic of the respective regions but are not unique to them. The Polish cultivars were harvested as a ripe fruit in July 2020, while the Cypriot tomatoes were harvested ripped in October 2020. Phenotypic representation of the four tomato cultivars from Poland (Russian Gold, Black Plum, Paschalne, Lima) and four tomato cultivars from Cyprus (Cherry F1, Commodo F1, F179, Torry F1) at fully ripe stage are presented in Figure 8. Due to potential influence of weather and climate conditions on ripening process, the data showing the average temperature (°C) and rainfalls (mm) in Poland and Cyprus 2020 is shown in Table S3 (Supplementary Table S3).

Tomato fruits at ready-to-eat stage (visual and morphological parameters used to select the fruits, according to Polish Norm PN-71/R-75356) were cut and immediately placed in liquid nitrogen, ground into fine powder using mortar and pestle, and stored at −80 °C. For each cultivar, the analyses were performed using three independent biological replicates, consisting of pooled tissue from three tomato fruits per replicate. For biochemical analysis, all samples were lyophilized using a Christ alpha 1-4 LO plus freeze dryer (pressure 0.055 mbar, temperature −55 °C). The lyophilized samples were then stored in a laboratory freezer until needed and to avoid loss of bioactive compounds.

### 4.2. Titratable Acidity (TA)

To examine the acidity of tomato fruits, 1g of homogenized fruit tissue was mixed with distilled water and heated up to boiling point. Next, mixture was cooled, transferred to a 25 cm^3^ flask and filled with water up to 25 cm^3^. Samples were mixed and filtered. The obtained extracts were titrated with 0.01 M NaOH (Chempur, Piekary Śląskie, Poland) solution until the pH of the examined samples reached 8.1. In addition, 2–3 drops of phenolphthalein were added to observe the colour change. The acidity was calculated as mg/100 g of product.

### 4.3. Lycopene and β-Carotene Content

To determine the content of lycopene and β-carotene in tomato fruits, to 0.1 g of plant material was homogenized with one mL of acetone–hexane mixture (4:6) (Chempur, Poland) [40]. Samples were centrifuged at 4 °C for 5 min (Eppendorf Centrifuge 5415 R). An aliquot was taken from the upper phase, and the absorbance was measured at 663, 645, 505, and 453 nm (TECAN, Infinite 200^®^ PRO, Tecan Austria GmbH, Grödig, Austria). The content of lycopene, β-carotene was expressed as expressed μg 100 g^−1^ F.W.

Pure lycopene and β-carotene standards (Sigma-Aldrich, St. Louis, MO, USA; distributed in Europe by Merck KGaA, Darmstadt, Germany) were used to validate the spectrophotometric method. Calibration curves were constructed at the respective wavelengths (453, 505, 645, and 663 nm) to ensure accurate quantification.

Calculation was provided according to the Nagata and Yamashita (1992) [41].

### 4.4. Antioxidant Capacity

Total antioxidant capacity was evaluated using two methods; Ferric Reducing Antioxidant Power (FRAP) assay and 2,2′-azino-bis(3- ethylbenzothiazoline-6-sulphonic acid) (ABTS). The plant extracts for FRAP assay were prepared as follows. 0.05 g of tomato tissue was grounded with 2 mL hexane (Sigma-Aldrich, USA) solution and extracts were left overnight at −20 °C. After incubation samples were centrifuged (Eppendorf Centrifuge 5415 R) for 10 min at 4 °C. Next, 400 μL of extract was mixed with 1.98 mL of FRAP reagent [1.65 mL of 0.3 Μ CH_3_COONa.3H_2_O (pH 3.6), 0.165 mL of 20 mM FeCl_3_*10H_2_O, 0.165 mL of 10 mM Tripyridil-s-triazine (TPTZ) (Sigma-Aldrich, USA) dissolved in 40 mM HCl)] [40]. Subsequently, 400 μL of Tween20 (Sigma-Aldrich, USA) was added to the sample and mixture was vigorously vortex. The samples were then incubated for 4 min at 37°C. The FRAP reagent was freshly prepared, and the absorbance was compared against the standard curve for quantification at 593 nm (TECAN, Infinite 200^R^ PRO).

The ABTS assay was conducted in accordance with the method described by Georgiadou et al. [40]. Extracts were prepared by mixing 0.05 g of tomatoes tissue with 2 mL 50% *v*/*v* acetone (Sigma-) and were placed at 4 °C for 24 h. Subsequently, samples were centrifuged for 10 min at 16,000 RCF× *g* at 4°C (Eppendorf Centrifuge 5415 R). Next, 50 μL of the extract was mixed with 1 mL of the freshly diluted ABTS+. The solution of 2,2′-azinobis (3-ethylbenzothiazoline-6-sulfonic acid) (Sigma-Aldrich, USA) radical cation (ABTS+.) was produced by the reaction of 7 mM ABTS water solution with 2.45 mM potassium persulfate (Sigma-Aldrich, USA) which was left in the dark at room temperature for 12–16 h before use. The samples were incubated for 1 min at 30 °C. The ABTS radical scavenging activity was calculated based on the percentage inhibition of absorbance at 734 nm relative to the Trolox standard curve (TECAN, Infinite 200^R^ PRO).

### 4.5. Profilin and Bet v 1 ELISA Analysis

To obtain protein from fruit, the Total Protein Extraction Kit for Plant Tissues was used according to manufacturer’s instructions (Sigma-Aldrich, USA). The allergenic potential was evaluated using an indirect, non-competitive ELISA based on the method of Hallmann et al. (2019) [42], with detailed modifications as described below. Tris-glycine protein extracts were first diluted tenfold in carbonate-bicarbonate buffer (pH 9.6; Sigma-Aldrich, USA). Aliquots of 100 µL were dispensed into 96-well MaxiSorp polystyrene microplates (Thermo Scientific; Nunc™, Roskilde, Denmark) in six technical replicates (three for test samples and three for corresponding negative controls). Plates were incubated overnight at 4 °C to allow protein binding, after which the wells were emptied and washed four times with phosphate-buffered saline (PBS; 137 mM NaCl, 2.7 mM KCl, 10 mM Na_2_HPO_4_, 1.8 mM KH_2_PO_4_, supplemented with 0.1% *v*/*v* Tween 20). To block non-specific binding sites, 100 µL of a 3% (*w*/*v*) skimmed milk solution in PBS was added to each well, and plates were incubated for 2 h at room temperature. Following removal of the blocking solution and four washing steps with PBS-T, 100 µL of primary antibody solution (mouse anti-Bet v 1 or rabbit anti-profilin; both diluted 1:1000 in PBS) was added to the wells. The plates were incubated for 2 h at room temperature to allow antigen–antibody interaction. After washing, 100 µL of alkaline phosphatase-conjugated secondary antibodies (anti-mouse IgG or anti-rabbit IgG, diluted 1:10,000 in PBS) was added, followed by 1 h incubation at room temperature. Plates were washed four additional times with PBS-T to remove unbound antibodies. For detection, 100 µL of p-nitrophenyl phosphate (pNPP; Sigma-Aldrich, used undiluted) was added as a chromogenic substrate for alkaline phosphatase and incubated for 30 min at room temperature in the dark. The enzymatic reaction was terminated by the addition of 100 µL of 3 M NaOH to each well. Absorbance was then measured at 405 nm using a Multiscan RC microplate reader (Thermo Labsystems, Vantaa, Finland). Quantification of allergen-related proteins was performed using standard curves prepared with purified Bet v 1 allergen (Indoor Biotechnologies, Inc., Charlottesville, VA, USA) and plant profilin (Sigma-Aldrich, Warsaw, Poland). The calibration curves covered the linear range of the assay, and the detection limit was defined as the lowest analyte concentration that could be distinguished from the blank, calculated as the mean absorbance of the blank plus three standard deviations.

### 4.6. RNA Extraction

Total RNA was extracted from three biological replicates for each tomato cultivar following the CTAB protocol [43]. Plant tissues were mixed with extraction buffer (2% CTAB, 2.5% PVP-40, 2 M NaCl 100 mM Tris-HCl pH 8.0, 25 mM EDTA pH 8.0 and 2% of β-mercaptoethanol added just before use). The samples were heated at 65 °C for 10 min and chloroform was added. The tube was mixed and centrifuged at 13.2 rpm for 10 min at 4 °C. The upper phase of supernatant was collected and mixed with LiCl (3 M final concentration). Samples were incubated at −20 °C overnight. After incubation, samples were centrifuged, supernatant was discarded and pellet was resuspended in pre-heated at 65 °C SSTE buffer (10 mM Tris–HCl pH 8.0, 1 mM EDTA pH 8.0, 1% SDS, 1 M NaCl). Following, chloroform was inserted, and tubes were centrifuged for 15 min. The upper phase of supernatant was transferred to new microcentrifuge tube and precipitated RNA was mixed with 2.5 volume of absolute ethanol and 1/10 volume of 3 M CH_3_COONa pH 5.2. Next, samples were incubated at −80 °C overnight. Following the incubation, pellet was washed with 75% (*v*/*v*) ethanol. Pellet was dried at block heater at 50 °C for three min and after that resuspended in sterilized ddH_2_O. To confirm the lack of DNA in prepared extracts, DNase treatment was applied with the use of DNase I (RNase free (Cat. No. NU01a, HT Biotechnology Ltd., Cambridge, UK), according to the procedure described in Georgiadou et al., 2016 [44].

### 4.7. cDNA Synthesis and Quantitative Real-Time RT-qPCR Analysis

To conduct cDNA synthesis, 1 μg of total RNA was reverse-transcribed using the Prime Script ^TM^ RT reagent Kit (Takara Bio, Kusatsu, Japan) according to the manufacturer’s instructions. Quantitative RT-PCR (qRT-PCR) was performed using a Biorad IQ5 real-time PCR cycler (Bio-Rad, Hercules, CA, USA). The reaction mix contained 4 μL cDNA in reaction buffer (5-fold diluted first-strand cDNA), 0.5 μL of each primer (10 pmol/mL) and 5 μL 2X master mix (KAPA SYBR^®^ FAST qPCR Kit, Kapa-Biosystems, Wilmington, MA, USA). The total reaction volume was 10 μL. The initial denaturation stage was at 95 °C for 5 min, followed by 40 cycles of amplification [95 °C for 30 s, annealing temperature (Tm 60–65 °C) for 30 s, and 72 °C for 30 s] and a final elongation stage at 72 °C for 5 min. Gene amplification cycle was followed by a melting curve run, carrying out 61 cycles with 0.5 °C increment between 65 and 95 °C. The nucleotides from the database ALLERGEN NOMENCLATURE WHO/IUIS Allergen Nomenclature Sub-Committee (http://www.allergen.org/index.php) (Accessed on 22 September 2020) (Supplementary Table S1) were used to design the primers with the Primer 3 (https://primer3.ut.ee/) (Accessed on 22 September 2020). The criteria considered in the selection and design of suitable primers were for instance, specificity, primers length, GC content. The annealing temperature of the primers used ranged between 60 and 62 °C (Supplementary Table S1). The *SlACT* gene was used as a housekeeping reference gene [45].

### 4.8. Statistical Analysis

The statistical analysis of real-time RT-qPCR results (pairwise fixed reallocation randomization test) was performed using the REST-XL software, according to Pfaffl et al. (2002) [46]. *SlACT* gene was used as a housekeeping reference gene and F179 cultivar were used for calibration of each respective cultivar. The biochemical analysis was performed using the software package SPSS v25.0 (SPSS Inc., Chicago, IL, USA) and the comparison of averages of each treatment was based on the analysis of variance (One-Way ANOVA) according to Duncan’s multiple range test at significance level 5% (*p* ≤ 0.05). The figures were generated using Prism 8.3.1 (GraphPad, La Jolla, CA, USA).

## 5. Conclusions

This study demonstrated that tomato fruit quality and allergenicity are jointly shaped by genetic background, ripening stage, and environmental conditions, with clear differences observed between Polish and Cypriot cultivars. Among the examined varieties, Torry F1 (Cypriot) stood out with the lowest levels of allergen gene expression, accompanied by reduced lycopene and β-carotene contents, suggesting a potentially lower allergenic risk for consumers. In contrast, Russian Gold (Polish) displayed selective up-regulation of *Sola l 3* genes despite reduced antioxidant activity, highlighting cultivar-specific regulatory differences. The Polish cultivars generally exhibited higher β-carotene levels, while Cypriot cultivars displayed stronger overall antioxidant activity, reflecting the influence of temperature and sunlight. These findings indicate that cultivars such as Torry F1 may be promising candidates for breeding programmes or consumer recommendations focused on reduced allergenicity, while Russian Gold and others require further evaluation due to inconsistent allergen expression patterns.

Methodologically, the comparison of analytical approaches revealed important discrepancies: while ELISA provided variable and sometimes contradictory results for allergen detection, RT-qPCR consistently captured gene expression patterns. Given the superior stability of nucleic acids over proteins and the reduced susceptibility to cross-reactivity, RT-qPCR emerges as a more reliable and precise method than ELISA for allergen analysis in tomato fruits. Recombinant plasmids containing target gene regions can further strengthen standardization and validation in future applications.

To summarize, these findings underline the importance of cultivar choice for nutritional quality and allergenic risk, while also supporting DNA/RNA-based approaches as the preferred strategy for allergen monitoring in tomato fruits. Practically, the results provide guidance for breeders selecting low-allergen cultivars, organic producers aiming to optimize fruit quality, and consumers seeking safer dietary options.

## Figures and Tables

**Table 1:** Titratable acidity (TA) of Polish tomatoes (Russian Gold, Black Plum, Paschalne, Lima) and Cypriot tomatoes (Cherry F1, Commodo F1, F179, Torry F1). Different letters indicate statistically significant differences (*p* < 0.05) when analyzed using Duncan’s multiple range test. Data represents the means of three biological replications ± SE.

Tomato Cultivars		TA (g 100 g^−1^ DW)
Polish	Russian Gold	0.039 ± 0.001 ^d^
	Black Plum	0.057 ± 0.001 ^c^
	Paschalne	0.067 ± 0.002 ^b^
	Lima	0.087 ± 0.001 ^a^
Cypriot	Cherry F1	0.013 ± 0.001 ^f^
	Commodo F1	0.018 ± 0.001 ^e^
	F179	0.055 ± 0.001 ^c^
	Torry F1	0.008 ± 0.001 ^g^

## Data Availability

The RNA-Seq data generated in this can be found in the GenBank (NCBI Sequence database). Accession numbers are obtained from Allergen Nomenclature, WHO/IUIS Allergen Nomenclature Sub-Committee (http://www.allergen.org/index.php (accessed on 23 September 2025)). *SlSola l 1* (AJ417553) [https://www.ncbi.nlm.nih.gov/gene/?term=AJ417553 (accessed on 23 September 2025)] *SlSola l 2* (AF465612) [https://www.ncbi.nlm.nih.gov/nuccore/AF465612 (accessed on 23 September 2025)] *SlSola l 3* (U81996) [https://www.ncbi.nlm.nih.gov/nuccore/U81996 (accessed on 23 September 2025)] *SlSola l 4* (KF682291) [https://www.ncbi.nlm.nih.gov/nuccore/KF682291 (accessed on 23 September 2025)] *SlSola l 5* (M55019) [https://www.ncbi.nlm.nih.gov/nuccore/M55019 (accessed on 23 September 2025)] *SlSola l 6* (NM_001319954.1) [https://www.ncbi.nlm.nih.gov/nuccore/NM_001319954.1 (accessed on 23 September 2025)] A housekeeping reference gene involved in this study can be found at the NCBI with the following accession numbers or gene IDs: *SlACT* (TC194780) Løvdal, T.; Lillo, C. Reference Gene Selection for Quantitative Real-Time PCR Normalization in Tomato Subjected to Nitrogen, Cold, and Light Stress. *Anal. Biochem.* **2009**, *387*, 238–242, doi:10.1016/j.ab.2009.01.024 [45].

## Supplementary Materials

The supporting information can be downloaded at: https://www.mdpi.com/article/10.3390/ijms26199446/s1.

## Abbreviations

The following abbreviations are used in this manuscript:

RT-qPCR	quantitative real-time polymerase chain reaction
qPCR	real-time polymerase chain reaction
FRAP assay	Ferric Reducing Antioxidant Power assay
ABTS assay	2,2’-azino-bis-(3-ethylbenzothiazoline-6-sulfonic) acid assay
*Sol l1*	profilin
*Sol l 2*	b-fructofuranosidase
*Sol l 3*	lipid transfer protein
*Sol l4*	intracellular pathogenesis-related protein
*Sol l 5*	cyclophilin
*Sol l 6*	7 kDa lipid transfer protein
*Sol l 7*	11 kDa lipid transfer protein
ELISA	Enzyme-Linked Immunosorbent Assay
TA	titratable acidity
RG	Russian Gold
BG	Black Plum
PAS	Paschalne
CH.	Cherry F1
COM	Commodo F1
TOR	Torry F1
