# Supplementary material for: Suppressed Expression of Allergenic Transcripts in Different Tomato Cultivars Are Linked with Increased Antioxidant Capacity
Source: Int J Mol Sci. 2025 Sep 27;26(19):9446. doi: 10.3390/ijms26199446 (PMC12524623; doi:10.3390/ijms26199446)
Supplement: Supplementary file 1 [file ijms-26-09446-s001.zip › ijms-3805108-supplementary.pdf]

Supplementary Table S1. Oligonucleotide primers used for real-time RT-qPCR analyses. Accession numbers are from Allergen Nomenclature, WHO/IUIS Allergen Nomenclature Sub-Committee (http://www.allergen.org/index.php). Previously published housekeeping reference gene (*ACT*) used.

Gene	Reference	Accession number	Primer	Nucleotide sequence 5'-3'	Tm (°C)
SlACT	**Løvdal and Lillo, 2009**	TC194780	*SlACT-For*	GAAATAGCATAAGATGGCAGACG	60
			SlACT-Rev	ATACCCACCATCACACCAGTAT	
SlSola l 1	-	AJ417553	*SlSolal1 -For*	ATTATTGGCCAAGATGGAACTG	60
			SlSolal1-Rev	GCCTGATTGGTCTTCTTAATGG	
SlSola l 2	-	AF465612	*SlSolal2-For*	TGCTTTCTGTAGCCTTCTTTCC	60
			SlSolal2-Rev	GAAAATGGTAAGCCGTTCTTTG	
SlSola l 3	-	U81996	*SlSola3-For*	GTTGTGGTGGTGTTAAGGGTCT	60
			SlSolal3-Rev	GAAGGGCTGATCTTGTAAGGAA	
SlSola l 4	-	KF682291	*SlSolal4v.1-For*	TGAGTCAACAACCACAATTTCC	60

			SlSolal4v.1-Rev	ATTGGACCACCTTCAACAAAGT	
SlSola l 5	-	M55019	*SlSolal5-For*	TGAAGGCATGGATGTGATTAAG	60
			SlSolal5-Rev	CCTAAAACGGCGACTCAAATAC	
SlSola l 6	-	NM_001319954.1	*SlSolal6-For*	AGGTTTGTTCTTTTGTGGCAGT	62
			SlSolal6-Rev	GTCCAGCAACTTTCCTAGCATT	

Supplementary Table S2. Relative expression levels of allergen genes (*SlSolal 1, SlSolal 2, SlSolal 3, SlSolal 4, SlSolal 5 and SlSolal 6*) of four tomato cultivars from Poland (Russian Gold, Black Plum, Paschalne, Lima) and four tomato cultivars from Cyprus (Cherry F1, Commodo F1, F179, Torry F1) (n = 3). Values that differ from the control (tomato cultivar 'F179' from Cyprus) with significance level P ≤ 0.05 are marked with bold letters. Data are based on a statistical analysis of the means of three biological replications (**Pfaffl et al., 2002**).

Tomato Cultivars		Genes					
		SlSolal 1	*SlSolal 2*	*SlSolal 3*	*SlSolal 4*	*SlSolal 5*	*SlSolal 6*
Polish tomatoes	Russian Gold (RG)	**3.701**	-1.234	**12.484**	**7.928**	**15.327**	1.049
	Black Plum (BP)	**9.481**	**18.507**	1.234	**3.598**	**20.043**	**3.732**
	Paschalne (PAS)	**6.998**	**11.020**	1.098	**11.385**	**13.343**	**12.143**
	Lima (L)	**3.147**	**12.755**	**3.837**	1.162	**8.404**	**1.781**
Cypriot tomatoes	Cherry F1 (CH)	1.507	**-1.793**	1.957	2.242	2.207	**2.418**
	Commodo F1 (COM)	**1.608**	**-1.331**	**2.898**	**3.633**	1.097	**-8.920**
	Torry F1 (TOR)	1.158	**-1.396**	-2.518	**-7.632**	-1.478	**-3.050**
**Tomato Cultivars**		**p-values**					
		SlSolal 1	*SlSolal 2*	*SlSolal 3*	*SlSolal 4*	*SlSolal 5*	*SlSolal 6*
Polish tomatoes	Russian Gold (RG)	**0.0010**	0.6370	**0.0010**	**0.0010**	**0.0010**	0.7815
	Black Plum (BP)	**0.0010**	**0.0010**	0.8860	**0.0010**	**0.0325**	**0.0415**
	Paschalne (PAS)	**0.0010**	**0.0010**	0.9050	**0.0395**	**0.0375**	**0.0455**
	Lima (L)	**0.0010**	**0.0010**	**0.0010**	0.6080	**0.0010**	**0.0455**
Cypriot tomatoes	Cherry F1 (CH)	0.1965	**0.0325**	0.6005	0.1290	0.2410	**0.0010**
	Commodo F1 (COM)	**0.0455**	**0.0325**	**0.0325**	**0.0180**	0.6555	**0.0010**
	Torry F1 (TOR)	0.5435	**0.0325**	0.2840	**0.0010**	0.1055	**0.0455**

Supplementary Table S3. Weather and climate data for Poland and Cyprus (2020) [19,20].

**Poland**												
**Month**	I	II	III	IV	V	VI	VII	VIII	IX	X	XI	XII
**Average temp. [°C]**	2.2	4.0	4.5	8.7	11.0	17.7	18.2	19.8	15.1	10.5	5.6	1.9
**Rainfalls**	645.4 mm											
**Cyprus**												
**Month**	I	II	III	IV	V	VI	VII	VIII	IX	X	XI	XII
**Average temp. [°C]**	16.6	17.1	19.4	22.3	27.4	27.9	31.8	32.5	32.2	29.5	23.6	20.6
**Rain falls**	397.3 mm											
